# Comparative Outcomes of Transabdominal Preperitoneal and Totally Extraperitoneal Laparoscopic Inguinal Hernia Repair: A Retrospective Cohort Study

**DOI:** 10.7759/cureus.104487

**Published:** 2026-03-01

**Authors:** Srikanth N Jarupla, Ganesh Vadthya, Ajit K Naik, Heena Dixit, Prashant Pillai, Anil Managutti, Seema Gupta

**Affiliations:** 1 Department of General Surgery, Dr BS Kushwah Institute of Medical Sciences, Kanpur, IND; 2 Department of General Surgery, Deccan College of Medical Sciences, Hyderabad, IND; 3 Department of General Surgery, Pandit Raghunath Murmu Medical College and Hospital, Baripada, IND; 4 Department of Blood Cell, Commissionerate of Health and Family Welfare, Hyderabad, IND; 5 Department of Oral and Maxillofacial Surgery, RKDF Dental College and Research Centre, Bhopal, IND; 6 Department of Oral and Maxillofacial Surgery, Narsinhbhai Patel Dental College and Hospital, Visnagar, IND; 7 Department of Orthodontics, Kothiwal Dental College and Research Centre, Moradabad, IND

**Keywords:** hernia, inguinal, laparoscopy, postoperative complications, retrospective study

## Abstract

Introduction: Laparoscopic inguinal hernia repair is widely performed using either the transabdominal preperitoneal (TAPP) or totally extraperitoneal (TEP) approach. Although both techniques are established, uncertainty persists regarding their relative perioperative and postoperative outcomes in routine clinical practice. The aim of the present study was to compare operative parameters, postoperative recovery, complication rates, and short-term outcomes between TAPP and TEP laparoscopic inguinal hernia repair.

Materials and methods: This retrospective cohort study included 100 adult patients who underwent elective laparoscopic inguinal hernia repair between August 2021 and April 2023. Patients were equally divided into TAPP (n = 50) and TEP (n = 50) groups. Demographic characteristics, operative details, postoperative pain, length of hospital stay, intraoperative conversion, complications, and follow-up outcomes were analyzed. Continuous variables were compared using independent samples t-tests, and categorical variables were analyzed using Fisher's exact or chi-squared tests.

Results: Baseline demographic and hernia-related characteristics were comparable between the groups. A significantly higher intraoperative conversion rate was observed in the TEP group. Operative time, postoperative pain scores, and hospital stay did not differ significantly between techniques. Rates of short-term complications, including seroma, infection, and urinary retention, were similar. Short-term outcomes, including recurrence and chronic groin pain, showed no statistically significant differences during follow-up.

Conclusion: Both TAPP and TEP laparoscopic inguinal hernia repairs demonstrated comparable safety and effectiveness. However, the higher conversion rate associated with TEP highlights its technical complexity. Technique selection should be guided by surgeon experience, patient factors, and institutional expertise.

## Introduction

Inguinal hernia repair is one of the most commonly performed general surgical procedures worldwide, with laparoscopic techniques increasingly preferred due to reduced postoperative pain, faster recovery, and earlier return to normal activities compared with open repair [1]. Among laparoscopic approaches, transabdominal preperitoneal (TAPP) and totally extraperitoneal (TEP) repairs are the two most widely practiced techniques, both based on mesh placement in the preperitoneal space but differing in surgical access and anatomical dissection [2].

The TAPP approach involves transperitoneal access, allowing the clear visualization of intra-abdominal anatomy and facilitating the identification of bilateral or occult hernias. However, it carries a potential risk of intra-abdominal complications related to peritoneal entry [3]. In contrast, the TEP approach avoids the violation of the peritoneal cavity and may reduce the risk of adhesions and visceral injury, though it is technically more demanding and associated with a steeper learning curve. Despite these differences, both techniques are considered safe and effective when performed by experienced surgeons [4,5].

Multiple studies have compared TAPP and TEP with respect to operative time, postoperative pain, complications, recurrence, and recovery outcomes [4,5]. However, the results remain inconsistent, and the choice of technique often depends on surgeon preference, institutional practice, and patient factors rather than clear evidence of superiority. Given the ongoing debate regarding the relative advantages of TAPP and TEP, further comparative evaluation is warranted to inform surgical decision-making and optimize patient outcomes. The aim of this retrospective study was to compare the clinical outcomes of laparoscopic inguinal hernia repair performed using the TAPP and TEP techniques. The specific objectives were to evaluate and compare operative parameters, postoperative pain, length of hospital stay, complication rates, and short-term outcomes between the two approaches.

## Materials and methods

Study design

This study was designed as a retrospective cohort analysis of patients who underwent laparoscopic inguinal hernia repair using either the TAPP or TEP technique from August 2021 to April 2023 in the Department of General Surgery, Deccan College of Medical Sciences, Hyderabad, India. The study protocol was reviewed by the Institutional Ethical Committee of Deccan College of Medical Sciences (approval number: 2023/61/065) and deemed exempt from full review. A waiver of informed consent was granted due to the retrospective analysis of anonymized medical records. Patient confidentiality was strictly maintained throughout the study. Data were anonymized at the time of extraction, and all identifiers were removed prior to analysis. The study was conducted in accordance with the ethical principles outlined in the Declaration of Helsinki and relevant institutional guidelines.

Sample size

A post hoc power analysis was performed to assess whether the available sample size provided adequate statistical power to detect a moderate standardized mean difference in primary continuous outcomes. Using the G*Power software (version 3.1; Heinrich Heine University Düsseldorf, Düsseldorf, Germany), a two-tailed independent samples t-test was selected as the primary model for sample size estimation. The analysis was parameterized to detect a medium effect size (Cohen's d = 0.50), based on the standardized mean differences for key operative or recovery outcomes reported in a prior study. With an alpha (α) level set at 0.05 (5% confidence) and a beta (β) level set at 0.20 (80% statistical power), the calculation yielded a minimum required sample size of 100 total participants (n = 50 per group). Assuming equal allocation, two-sided test, significance level α, and power 1−β1, the sample size formula used was as follows: $n = \frac{2\left(Z_{\alpha/2} + Z_{\beta}\right)^2 \sigma^2}{\Delta^2}$. Here, n is the sample size per group, Zα/2​ is the critical value of the standard normal distribution (Z​ = 1.96 for α = 0.05), Zβ is the critical value for power (Z = 0.84 for 80% power), σ is the pooled standard deviation of the outcome, and Δ is the clinically meaningful difference in means.

Study population

One hundred adult patients diagnosed with inguinal hernia and managed surgically by laparoscopic repair were eligible for inclusion. Patients were categorized into two groups based on the surgical technique employed: the TAPP group (n = 50) and the TEP group (n = 50). Patients aged 18 years and above who underwent elective laparoscopic inguinal hernia repair with mesh placement and had complete perioperative and follow-up records with a minimum follow-up of six months were included in the study. Both unilateral and bilateral primary inguinal hernias were considered. Patients were excluded if they had recurrent inguinal hernia, complicated hernias (incarcerated or strangulated), emergency surgeries, or incomplete clinical documentation. Patients lost to follow-up before the minimum required duration were also excluded from outcome analysis. The choice of surgical technique (TAPP or TEP) was determined by the operating surgeon based on individual clinical judgment, surgeon expertise, and intraoperative considerations. Factors influencing technique selection included surgeon preference, prior abdominal surgery, hernia characteristics, and anticipated technical complexity. No formal randomization or standardized allocation protocol was used.

Surgical technique

All procedures were performed under general anesthesia by surgeons experienced in laparoscopic hernia repair. In the TAPP technique, access to the preperitoneal space was achieved via transperitoneal entry, followed by mesh placement and peritoneal closure. In the TEP technique, the preperitoneal space was developed without breaching the peritoneal cavity, and the mesh was positioned directly over the myopectineal orifice. A standard lightweight polypropylene flat mesh (approximately 10 × 15 cm) was used in both TAPP and TEP repairs according to institutional protocol. In TAPP repairs, mesh fixation was performed selectively using absorbable tacks, particularly in large direct defects, whereas in TEP repairs, mesh was placed without routine fixation unless deemed necessary by the operating surgeon. The fixation strategy was consistent across groups and guided by hernia characteristics and intraoperative assessment. Peritoneal closure was performed using a continuous absorbable non-barbed suture as per institutional protocol. Tack or glue-based peritoneal closure methods were not used. Postoperative analgesia was standardized across both groups. All patients received scheduled non-steroidal anti-inflammatory drugs (NSAIDs) and intravenous/oral acetaminophen, with opioids administered on an as-needed basis for breakthrough pain. Local anesthetic infiltration at port sites was performed at the end of the procedure. No routine regional nerve blocks were used.

Data collection

Demographic data including age, sex, body mass index (BMI), and comorbidities were recorded. Hernia-related variables such as laterality (unilateral or bilateral) and hernia type were documented. Operative details included the duration of surgery and intraoperative complications. Postoperative data were extracted from inpatient records and follow-up visits.

Outcome measures

Primary outcome measures included operative time, postoperative pain scores assessed using a visual analog scale (VAS), length of hospital stay, and early postoperative complications such as seroma, hematoma, surgical site infection, and urinary retention. Secondary outcomes included chronic groin pain, recurrence rate, and time to return to normal daily activities. Chronic groin pain was assessed during routine outpatient follow-up through direct clinical interview and pain scoring using a 10-point VAS. Pain persisting beyond three months postoperatively was classified as chronic. The assessment evaluated pain intensity and its impact on routine daily activities. A validated pain questionnaire was not routinely used, and neuropathic pain characteristics (such as burning sensation, paresthesia, and allodynia) were not systematically recorded. Patients were routinely followed up at standard postoperative intervals as per institutional protocol. Clinical records from outpatient visits were reviewed to assess postoperative recovery, complications, and recurrence. Intraoperative conversion was defined as conversion from the planned laparoscopic repair to open inguinal hernia repair. Conversion from TEP to TAPP or addition of extra ports was not performed in this cohort.

Statistical analysis

Data were analyzed using standard statistical software (IBM SPSS Statistics for Windows, V. 22.0 (IBM Corp., Armonk, NY, USA)). Continuous variables were expressed as mean ± standard deviation or median with interquartile range, depending on data distribution, while categorical variables were expressed as frequencies and percentages. Comparative analysis between the TAPP and TEP groups was performed using the independent samples t-test for continuous variables and Fisher's exact test for categorical variables. A p-value of <0.05 was considered statistically significant.

## Results

The median follow-up duration was nine months (interquartile range (IQR): 6-14 months; range: 6-22 months). The TAPP (n = 50) and TEP (n = 50) groups were well-matched at baseline. The absence of significant demographic or clinical differences between the two surgical cohorts at the study's outset indicates successful group comparability. This baseline equivalence strengthens the internal validity of the study, allowing for a more confident attribution of any observed differences in postoperative outcomes to the surgical technique (TAPP vs. TEP) itself, rather than to confounding pre-existing patient characteristics (Table 1). 

**Table 1:** Baseline demographic and clinical characteristics of the study population. Continuous variables were analyzed using the independent samples t-test and are presented as mean ± standard deviation (SD). Categorical variables were analyzed using the chi-squared test and are presented as numbers (percentages). A p-value of >0.05 denotes no statistical significance. BMI: body mass index

Characteristic		Total cohort (n = 100)	TAPP group (n = 50)	TEP group (n = 50)	Test statistic	P-value
Age (years)	Mean ± SD	49.2 ± 11.8	47.8 ± 12.5	50.6 ± 11.0	-0.85	0.398
Sex, n (%)	Male	94 (94%)	48 (96%)	46 (92%)	0.35	0.555
BMI (kg/m²)	Mean ± SD	26.1 ± 3.3	25.8 ± 3.5	26.4 ± 3.1	-0.64	0.523
Hernia laterality, n (%)	Unilateral	70 (70%)	34 (68%)	36 (72%)	0.00	1.000
	Bilateral	30 (30%)	16 (32%)	14 (28%)		
Hernia type, n (%)	Indirect	58 (58%)	30 (60%)	28 (56%)	0.24	0.888
	Direct	36 (36%)	16 (32%)	20 (40%)		
	Combined	6 (6%)	4 (8%)	2 (4%)		

A statistically significant difference was observed in the intraoperative conversion rate, favoring the TAPP technique (p = 0.030). For other early outcomes, operative time, postoperative pain, and length of stay, the observed mean differences favored TAPP but were not statistically significant, with confidence intervals indicating our data remain compatible with no true difference or even a small benefit for TEP. However, the clinically meaningful trend of a higher conversion rate with TEP aligns with larger-scale evidence, indicating it may present a greater technical challenge. The lack of statistical significance is likely due to the study's limited sample size, which is underpowered to detect differences in such low-frequency events (Table 2).

**Table 2:** Intraoperative and early postoperative outcomes. ^†^Owing to a zero-cell count; *p < 0.05 denotes statistical significance. RR was calculated with continuity correction. CIs were calculated using Koopman's score method. Mean differences were analyzed using the independent samples t-test. Categorical variables were analyzed using Fisher's exact test (two-sided). MD: mean difference; RR: relative risk; CI: confidence interval; VAS: visual analogue scale

Outcome measures		TAPP group (n = 50)	TEP group (n = 50)	Effect estimate (95% CI)	P-value
Operative time (min)	Mean ± SD	65.4 ± 18.2	70.8 ± 20.1	MD: -5.40 (-16.04 to 5.24)	0.314
Intraoperative conversion	n (%)	0 (0%)	6 (12%)	RR: 0.08^†^ (0.00 to 1.33)	0.030*
Pain VAS score (day 1)	Mean ± SD	3.5 ± 1.3	3.8 ± 1.4	MD: -0.30 (-1.07 to 0.47)	0.435
Length of hospital stay (days)	Mean ± SD	1.1 ± 0.5	1.4 ± 0.6	MD: -0.30 (-0.62 to 0.02)	0.064

Analysis of short-term complications revealed no statistically significant differences between the TAPP and TEP groups. Rates of seroma/hematoma, surgical site infection, urinary retention, and visceral/vascular injury were comparable, with all p-values >0.05. While point estimates for complications like seroma and urinary retention favored the TAPP technique, none reached statistical significance. The 95% confidence intervals for all outcomes were exceptionally wide, indicating our data are compatible with a clinically meaningful reduction in risk with TAPP, a meaningful increase in risk, or no true difference. The study is underpowered to draw definitive conclusions about complication rates (Table 3). No peritoneal closure-related complications, including peritoneal dehiscence, bowel obstruction, or port-site hernia attributable to closure technique, were observed during follow-up.

**Table 3:** Short-term postoperative complications. ^†^Due to a zero-cell count. RR was calculated with continuity correction. CIs were estimated using Koopman's asymptotic score. All p-values were derived from two-sided Fisher's exact tests. A p-value of >0.05 denotes no statistical significance. RR: relative risk; CI: confidence interval

Complication	TAPP group (n = 50)	TEP group (n = 50)	RR	95% CI	P-value
Seroma or hematoma, n (%)	6 (12%)	10 (20%)	0.60	0.24-1.53	0.285
Surgical site infection, n (%)	2 (4%)	4 (8%)	0.50	0.10-2.61	0.677
Urinary retention, n (%)	2 (4%)	6 (12%)	0.33	0.07-1.57	0.264
Visceral/vascular injury, n (%)	0 (0%)	2 (4%)	0.20^†^	0.01-4.06	0.495

Point estimates for key long-term outcomes, including hernia recurrence and chronic pain, favored the TAPP technique. However, the confidence intervals for all effect estimates were exceedingly wide, crossing the line of no effect. This indicates that while our data are consistent with a potential clinical benefit for TAPP, they are also compatible with a benefit for TEP or no true difference. The study lacks the precision to draw definitive conclusions regarding these secondary outcomes (Table 4).

**Table 4:** Secondary outcomes during follow-up. ^†^Owing to a zero-cell count. RR was calculated with continuity correction. CI was calculated with Koopman's asymptotic score method. Categorical variables were analyzed using Fisher's exact or chi-squared tests, as appropriate. Continuous variables were analyzed using the independent samples t-test. A p-value of >0.05 denotes no statistical significance. RR: relative risk; MD: mean difference; CI: confidence interval

Outcome measures		TAPP group (n = 50)	TEP group (n = 50)	Test value (95% CI)	P-value
Hernia recurrence	n (%)	0 (0%)	2 (4%)	RR: 0.20^†^ (0.01-4.06)	0.495
Chronic pain (≥6 months)	n (%)	2 (4%)	6 (12%)	RR: 0.33 (0.07-1.58)	0.164
Time to return to work (days)	Mean ± SD	13.5 ± 4.1	14.6 ± 5.3	MD: -1.10 (-3.78 to 1.58)	0.413

## Discussion

The present retrospective comparison of laparoscopic inguinal hernia repair techniques provides further insight into the relative performance of TAPP and TEP approaches in routine clinical practice. While both techniques demonstrated comparable perioperative safety and effectiveness, several clinically relevant trends emerged that merit discussion in light of existing evidence.

One of the most notable observations was the higher intraoperative conversion rate associated with the TEP approach. This finding aligns with multiple prior studies reporting that TEP, although conceptually attractive due to avoidance of the peritoneal cavity, is technically more demanding and associated with a steeper learning curve [2,6,7]. Large registry-based analyses and randomized trials have consistently shown higher conversion rates during the early phases of surgeon experience with TEP, primarily due to limited working space, peritoneal breaches, and difficulty in anatomical orientation [8]. In contrast, the TAPP approach offers a wider operative field and clearer visualization of key anatomical landmarks, which may explain the lower likelihood of conversion observed in both the present study and earlier reports [8].

Operative time, postoperative pain scores, and length of hospital stay were broadly comparable between the two techniques. Previous studies have reported conflicting results regarding operative duration, with some suggesting longer operative times for TEP due to technical complexity [9,10], while others demonstrate equivalence once surgeon experience is accounted for [11]. The absence of a significant difference in postoperative pain and hospital stay in the present study is consistent with contemporary literature suggesting that both techniques offer similar short-term recovery profiles when performed by experienced surgeons using standardized perioperative protocols [8,10].

The analysis of short-term postoperative complications revealed no meaningful differences between TAPP and TEP. This finding corroborates previous studies demonstrating comparable rates of seroma, hematoma, surgical site infection, and urinary retention between the two approaches [11,12]. Bansal et al. [13] reported higher early postoperative pain with TAPP. Although TAPP involves transperitoneal access, concerns regarding increased risk of visceral injury or intra-abdominal complications have not been substantiated in large-scale studies, particularly when meticulous surgical technique and proper peritoneal closure are employed. Conversely, while TEP theoretically reduces intra-abdominal risk, its restricted working space may predispose to peritoneal tears and procedural difficulty, particularly during the learning phase. Krishna et al. [3] reported higher postoperative pain and a higher incidence of scrotal edema in TAPP.

Short-term outcomes, including hernia recurrence and chronic groin pain, also did not differ statistically significantly between the two groups. This finding is in agreement with long-term follow-up data from a registry study indicating that recurrence rates after laparoscopic mesh repair are more closely related to mesh positioning, fixation technique, and surgeon expertise than to the choice of TAPP or TEP per se [14]. Chronic pain following laparoscopic inguinal hernia repair remains a multifactorial issue influenced by nerve handling, mesh characteristics, and fixation methods. The comparable incidence observed in the present study reinforces the view that both approaches are acceptable from a short-term functional standpoint. In contrast, Gass et al. [15] reported a higher incidence of intraoperative complications, longer operative time, and shorter hospital stay in patients undergoing the TAPP technique. Taken together, the results support the prevailing consensus that neither TAPP nor TEP is universally superior. Instead, the choice of technique should be individualized based on surgeon expertise, patient characteristics, and institutional resources. The higher conversion rate observed with TEP in this cohort highlights the importance of adequate training and case selection, particularly in centers with variable surgeon experience.

While multiple randomized controlled trials and meta-analyses have demonstrated broadly comparable outcomes between TAPP and TEP in terms of operative time, postoperative pain, complications, and recurrence, important uncertainties remain. Specifically, prior evidence shows variability in intraoperative conversion rates, inconsistent reporting of chronic groin pain, and limited power to detect differences in low-frequency outcomes such as recurrence. Moreover, learning curve effects and surgeon experience are frequently acknowledged but insufficiently quantified in real-world, non-trial settings. The most consistent finding across high-level evidence is that neither technique is universally superior and outcomes are strongly influenced by surgeon expertise rather than the approach itself. Our cohort uniquely contributes real-world data from a routine tertiary care setting with standardized institutional protocols but variable surgeon experience, thereby highlighting conversion risk and practical performance outside strictly controlled randomized controlled trial environments. 

Clinical implications

From a clinical perspective, the findings suggest that TAPP may offer greater procedural reliability, especially in settings where surgeon experience with TEP is limited or in patients with challenging anatomy. TEP remains a valid and effective option in experienced hands and may be preferred in patients where avoidance of peritoneal entry is desirable. Importantly, the overall similarity in postoperative recovery, complication rates, and long-term outcomes underscores that both techniques can be safely offered, provided appropriate expertise is available.

Limitations

This study has several limitations that should be acknowledged. First, its retrospective design inherently limits causal inference and introduces the possibility of selection bias, as the choice of surgical technique was based on surgeon preference rather than randomization. Second, the sample size was relatively small, limiting statistical power, particularly for infrequent outcomes such as recurrence and visceral injury. The study cohort was overwhelmingly male, reflecting the epidemiology of inguinal hernia; however, this limits the generalizability of the findings to female patients. The small number of female cases precluded meaningful sex-specific subgroup analysis. Third, follow-up duration, although sufficient to assess early recurrence and chronic pain, may be inadequate to capture late recurrences. Recurrence following laparoscopic inguinal hernia repair can occur beyond one year, and pain outcomes may evolve over time. Therefore, long-term durability cannot be definitively established from the present data. Finally, data on surgeon experience, mesh fixation methods, and postoperative analgesic protocols were not uniformly available, which may have influenced outcomes. 

The retrospective allocation of surgical technique based on surgeon preference introduces potential selection bias. Although all procedures were performed by surgeons experienced in laparoscopic hernia repair, surgeon-specific case volumes, learning curve stages, and technique-specific expertise were not uniformly available in the medical records. Therefore, the higher conversion rate observed in the TEP group may partly reflect differential surgeon experience rather than intrinsic technical differences between approaches. Residual confounding cannot be excluded.

## Conclusions

Both TAPP and TEP laparoscopic techniques are safe and effective options for elective inguinal hernia repair, with comparable postoperative recovery, complication rates, and short-term outcomes. TEP may be associated with greater technical demands, particularly in settings with variable surgeon experience. In routine clinical practice, TAPP may offer greater procedural reliability, particularly in centers with variable expertise or during the early learning curve. Ultimately, technique selection should be individualized, guided by surgeon proficiency, patient anatomy, and institutional resources to optimize surgical outcomes.
